# Vertical decomposition with Genetic Algorithm for Multiple Sequence Alignment

**DOI:** 10.1186/1471-2105-12-353

**Published:** 2011-08-25

**Authors:** Farhana Naznin, Ruhul Sarker, Daryl Essam

**Affiliations:** 1 School of Engineering and Information Technology, University of New South Wales at Australian Defence Force Academy, Canberra, Australia

## Abstract

**Background:**

Many Bioinformatics studies begin with a multiple sequence alignment as the foundation for their research. This is because multiple sequence alignment can be a useful technique for studying molecular evolution and analyzing sequence structure relationships.

**Results:**

In this paper, we have proposed a Vertical Decomposition with Genetic Algorithm (VDGA) for Multiple Sequence Alignment (MSA). In VDGA, we divide the sequences vertically into two or more subsequences, and then solve them individually using a guide tree approach. Finally, we combine all the subsequences to generate a new multiple sequence alignment. This technique is applied on the solutions of the initial generation and of each child generation within VDGA. We have used two mechanisms to generate an initial population in this research: the first mechanism is to generate guide trees with randomly selected sequences and the second is shuffling the sequences inside such trees. Two different genetic operators have been implemented with VDGA. To test the performance of our algorithm, we have compared it with existing well-known methods, namely PRRP, CLUSTALX, DIALIGN, HMMT, SB_PIMA, ML_PIMA, MULTALIGN, and PILEUP8, and also other methods, based on Genetic Algorithms (GA), such as SAGA, MSA-GA and RBT-GA, by solving a number of benchmark datasets from BAliBase 2.0.

**Conclusions:**

The experimental results showed that the VDGA with three vertical divisions was the most successful variant for most of the test cases in comparison to other divisions considered with VDGA. The experimental results also confirmed that VDGA outperformed the other methods considered in this research.

## Background

Multiple Sequence Alignment (MSA), the simultaneous alignment among three or more nucleotide or amino acid sequences, is one of the most essential tools in molecular biology. The goal of multiple sequence alignment is to align sequences according to their evolutionary relationships. For small lengths and small numbers of sequences, it is possible to create the alignment manually. However, efficient algorithms are essential for good alignments with more than eight sequences [1]. The existing algorithms can be classified into three main categories, exact, progressive and iterative. The iterative approaches can be of two types: iterative, and iterative plus stochastic.

The Dynamic Programming (exact method) approach [2] is good at finding the optimal alignment for two sequences. However, the complexity of this method grows significantly for three or more sequences [3]. We must mention here that MSA is a well-known combinatorial problem (NP-hard) where the computational effort becomes prohibitive with a large number of sequences [4].

The progressive alignment algorithm (Tree Based algorithm), proposed by Feng and Doolittle [5] iteratively utilizes the method of Needleman and Wunsch [2] in order to obtain an MSA and to construct an evolutionary tree to depict the relationship between sequences. A good number of global alignment algorithms, based on the progressive alignment method, have been proposed, such as MULTALIGN [6], MULTAL [7], PILEUP [8] and CLUSTALX [9]. MULTAL generates the final alignments by aligning closest sequences subsequently. MULTALIGN and PILEUP make the final alignments from the guide tree (the progressive alignment), which is constructed using UPGMA [10]. CLUSTAL W [1], based on a progressive approach, is a global method. The CLUSTAL W builds up the final alignments from a guide tree, which is calculated by a neighbour-joining algorithm [11]. CLUSTAL W uses the Weighted Sum of Pair score, which considers sequence weighting and position dependent gap penalties. Although this approach is successful in a wide variety of cases, it suffers from its greediness [12]. The CLUSTAL series (X, W and V) were developed based on the same algorithm. The alignments produced by these programs are exactly the same; the only difference among them is the way the user interacts with the program. PIMA [13] uses local dynamic programming to align only the most conserved motifs. It offers two alignments, MLPIMA and SBPIMA. T-Coffee [12] is a sensitive progressive alignment algorithm which combines information from both global and local alignments. This method is fast but there is a possibility to be trapped at a local minima. The difficulty with the progressive approach is that they usually converge to local optima [1]. To overcome such a limitation, it is recommended to use an iterative or stochastic procedure [14-16].

The iterative approach starts with an initial solution and then the current solution is improved using iterative steps. MUSCLE [17] is based on a progressive and iterative algorithm. It has three stages: draft progressive, improve progressive, and refinement. In each stage, a multiple sequence alignment is generated. Similarly MAFFT [18] is also based on a progressive and iterative algorithm. It uses a Fast Fourier Transform (FFT) to identify homologous regions. To evaluate the multiple sequence alignments, MAFFT uses the CLUSTAL W scoring system. ProbCons [19] is a Probabilistic and Consistency based algorithm. It computes posterior-probability matrices and expected accuracies for each pairwise comparison. It also computes an expected accuracy guide tree to progressively generate a final alignment by applying a probabilistic consistency transformation. ProbCons achieves more accurate results than MUSCLE and MAFFT, but is slower than those algorithms [19]. PRRP [20] is another global alignments program which is based on a progressive approach. This approach is robust, but it does not guarantee optimum solutions [12]. DIALIGN [21] uses a local alignment approach based on a segment to segment comparison, rather than on a residue to residue comparison. This method is successful in highly conserved flanking core blocks, but is unreliable outside the conserved motifs.

There are some iterative and stochastic approaches for MSAs (for example simulated annealing [22,23] and evolutionary computation [24-28]). HMMT [29], based on a simulated annealing method, maximizes the probability for sequence alignment where the solution could be trapped in local optima [30]. Evolutionary Algorithms (EAs) are population based stochastic global search algorithms. EA starts with an initial population of individual solutions. Different EAs use different representations (e.g. lists, trees, graphs) for the individuals and different reproduction operators (recombination or crossover and mutation) to generate offspring for the next generation. The main driving force in EAs is the selection of individuals based on their fitness (it may be based on the objective function, or some other kind of quality measure). Individuals with higher fitness have a higher probability to be chosen as members of the population of the next generation (or as parents for the generation of new individuals). This corresponds to the principle of survival of the fittest in natural evolution. There has been a variety of different EAs proposed over the years, such as Evolution strategies (ES), Evolutionary Programming (EP), genetic algorithms (GA) and their variants. GAs, the most well known algorithm in the EA family, have been successfully used for both numerical and combinatorial optimization. When using EAs for MSA, an initial seed is generated by a progressive alignment method, and then the steps of an EA are applied to improve the similarities among the sequences. For example, MSA-EA [31] improves the solution of the Clustal V [32] algorithm by initially generating one seed with Clustal V. This method works well when there are a large number of fully matched blocks, but performs poorly when there are only a few fully matched blocks [30].

There are other Genetic Algorithm (GA) based methods, such as SAGA [28], MSA-EC [33], GA-ACO [30], MSA-GA [34] and RBT-GA [35]. In SAGA, the initial generation is generated randomly with gap (null) symbols inserted randomly inside the sequences to make them equal in length. In this algorithm, 22 different operators are used to gradually improve the fitness of the MSA. These operators are dynamically scheduled during the evolution process. The time complexity of SAGA is large, mainly due to the time required by the repeated use of the fitness function [33]. Shyu *et al.*, [33] proposed two other approaches using GAs. In the first approach, GA was used to evolve an optimal guide tree which was created with the neighbor-joining method. Shyu's second approach facilitates the optimization of a consensus sequence with a GA by using a vertically scalable encoding scheme, in which the number of iterations needed to find an optimal solution is approximately the same regardless of the number of sequences being aligned. Another algorithm, GA-ACO [30], combines ant colony optimization with GA to overcome the problem of becoming trapped in local optimum. To do this, first GA is run with a randomly generated initial population (initial parent alignments). Its crossover operator then produces one or two offspring [31] from two parents, and a mutation operator provides another possible variation of the alignments. Finally, ACO (Ant Colony Optimization) was applied on the best alignment of the GA approach. MSA-GA is a simple GA based method with a different scoring function. To test this algorithm, the authors performed two sets of five runs for each of 28 test cases from the BAliBase 2.0 [36] dataset. RBT-GA combines GA with the Rubber Band Technique (RBT) to find optimal protein sequence alignments [37,38]. RBT is an iterative algorithm that uses a DP table. The authors [35] solved 34 problems from references 2 and 3 of the benchmark BAlibase 2.0 dataset. The experimental results showed that the overall performance of RBT-GA was better than the other methods compared in that paper. We must mention here that local search methods are sometimes integrated with GAs [39-43] to enhance the performance of GAs in solving MSA problems.

The EAs have an important advantage over progressive methods in that the alignment component can be made independent of the objective function. This means that different fitness functions can be tested without making any adjustment to the alignment procedure, which makes them particularly attractive for testing new objective functions. Another useful advantage of these methods is that the computational duration can be compressed by parallelization. These advantages motivate us to apply EAs to solve MSA problems in this research. In this paper, we propose a new approach based on a genetic algorithm, namely VDGA, where we introduce a decomposition method to divide the sequences into smaller subsequences. These subsequences are then processed separately before being combined back into whole sequences. We have used the guide tree method to generate an initial population, in each separate part of the decomposition, and also during the mutation. The proposed method starts with the DP distance table. In the DP distance table, the distance between two sequences is calculated from a pairwise alignment using Dynamic Programming (DP). We have used this distance table to generate a guide tree. We have applied and analyzed two techniques on the guide tree so as to generate an initial population. In running the GA, we have considered the Weighted Sum of Pair score as the fitness measure, with the PAM250 [44] score matrix and the CLUSTAL W default gap penalties.

The performance of the proposed algorithm has been compared with the state of the art GA and non-GA based methods, namely SAGA, MSA-GA, RBT-GA, PRRP, CLUSTALX, Clustal W, DIALIGN, HMMT, SB_PIMA, ML_PIMA, MULTALIGN and PILEUP8. To allow us to compare with other methods, we have calculated the corresponding BAliscore of the best WSPM score. For comparison, the results of the 26 datasets solved by MSA-GA, and the alignment results of the 34 datasets solved by RBT-GA were taken from the published papers [34] and [35] respectively. However, the results of the other methods mentioned above, were obtained from BAliBase 2.0 [36]. Based on the calculated BAliscores, VDGA outperforms the GA and non-GA based methods mentioned earlier.

This paper is organized as follows. After this background, the next section describes briefly the steps of a basic Genetic Algorithm and the details of the proposed VDGA method. The analyses of VDGA and comparisons with other well known methods have been discussed in the results and discussion section. Finally, we have summarised our work and planed for future work in the conclusions section.

## Methods

### Genetic Algorithms: The Basic structure

The general steps of the common GA can be summarized by the following pseudo-code:

1. Initialisation: Generate Initial population

2. Evaluation: Evaluate the individuals using a fitness function

3. Select individuals (parents) and then

4. Apply the genetic operators to them so as to create children

5. New generation: Create new generation from some combination of old generation and new child generations.

6. Go to 2 until it meets the stopping criteria_._

7. End.

### VDGA: The Proposed Algorithm

The proposed Vertical Decomposition using Genetic Algorithm (VDGA) is a modification of the basic GA. Its steps are: generation of initial population, generation of child population by applying genetic operators, forming a new population for the next generation, Vertical Division, and the stopping criteria. The Vertical Division is the key concept of the proposed algorithm. This technique is applied after generating the first/initial generation and after generating child generation. To do this, inside the Vertical Division step, the alignment is separated into different parts, the null symbols are removed from each part and then the tree-base method is applied on each part. After applying the tree-base method, each part produces an alignment. The alignments of each part are then combined to create a new alignment. As the solution of the progressive alignment method (guide tree) usually converges to a local optimum, therefore in the initial generation stage, we use the guide tree method to find the local optima and its neighbouring points in two ways; randomly generated subtree and shuffling. With the genetic operator (mutation), we also use the guide tree method. To calculate the guide tree, we use both DP [2] and the Kimura distance [45] tables as discussed below.

### Distance Calculation

#### Dynamic Distance Calculation

For this, the DP distance of each pair is calculated using equation (1) from a pairwise alignment [2]. To construct the DP distance matrix (table), which shows the distance between all sequence pairs are calculated,

(1) $$\text{Dynamic distance}=\left(mismatch\right)∕\left(align\;length\right)$$

#### Kimura Protein Distance Calculation

Equation (2) is an alternate means to calculate the distance, and was developed based on the relationship between the observed amino acid substitutions and the actual (corrected) substitutions from PAM or BLOSUM [46]. The match score is calculated by summing the number of exact matches. In this method, the partial matches between ambiguous symbols also contributes to the match score as fractional scores. The value of *S *is computed by dividing the match score by the number of positions scored. Gap positions are ignored, and only exact matches contribute to the match score [47].

(2) $$\begin{array}{c} \text{S}=\left(exact\text{\_}matches\right)∕\left(positions\text{\_}scored\right)\\ D=\text{1}-S\\ \text{Distance}=-ln\left(\text{1}-D-0.\text{2}D^{\text{2}}\right) \end{array}$$

**Figure 1:**
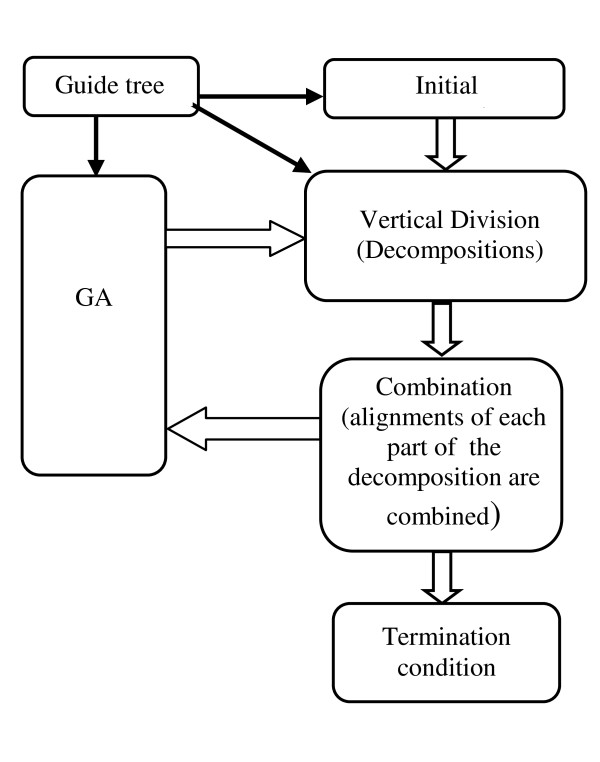
**Flowchart of VDGA**.

The flowchart of the proposed VDGA method is shown in Figure 1. In this figure, the block arrows represent the steps of the algorithm and the black color arrows represent the use of guide trees inside the proposed algorithm. The steps of this method are explained below.

### Initial Generation

**Figure 2:**
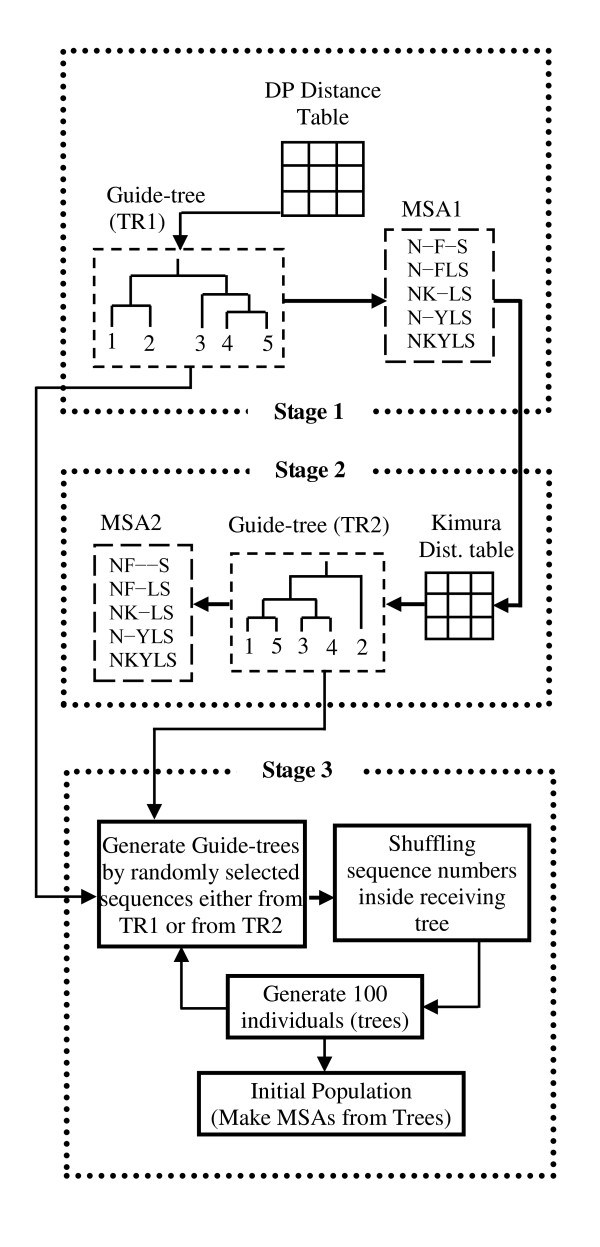
**Flowchart of Initial Generation**.

The aim of this step is to generate good initial solutions. The flowchart for generating the initial population is shown in Figure 2 and the stages are described below.

#### Stage 1

VDGA starts with a DP distance table. The guide tree is constructed from this table, which is referred to as TR1, and we generate a multiple sequence alignment (MSA1) from this guide tree.

#### Stage 2

In the second stage, the distance table is calculated from the multiple sequence alignment (MSA1), which is called the Kimura distance table. The Kimura distances are calculated from the aligned sequences. The second tree, TR2, is constructed from the Kimura distance table, and we then produce MSA2 as shown in Figure 2.

#### Stage 3

In this stage, two mechanisms are implemented on the two trees (TR1 and TR2) to generate 100 different trees. The first mechanism is to generate guide trees with randomly selected sequences and the second is shuffling the sequences inside those trees. The initial population produced by this method contains a set of multiple sequence alignments. Therefore, after receiving the set of guide trees, it needs to make a set of multiple sequence alignments. The functions of these mechanisms are explained below.

#### Mechanism 1

**Figure 3:**
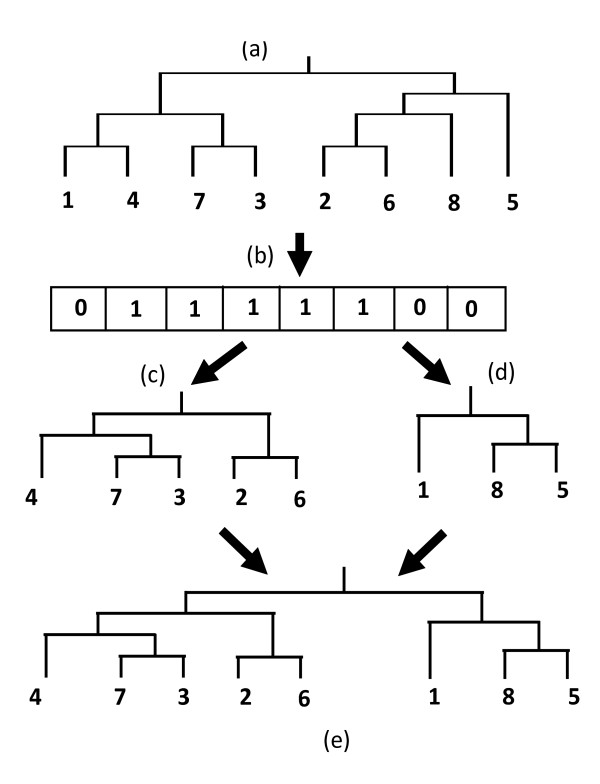
**(a) Guide tree; (b) 1 represents randomly selected sequence numbers from (a), and 0 represents unselected sequence numbers; (c) subtree made by the selected sequences; (d) subtree made by the remaining sequences of (a); and (e) new Guide tree made from (c) and (d)**.

In this case, sequence numbers are selected randomly from one tree (either TR1 or TR2). The selected sequences then make a new sub-tree with the same branching orders as the original one, and the non-selected sequences make another new sub-tree. Lastly, these two sub-trees are connected together to make a new tree. Figure 3 shows the behaviour of Mechanism 1.

#### Mechanism 2

**Figure 4:**
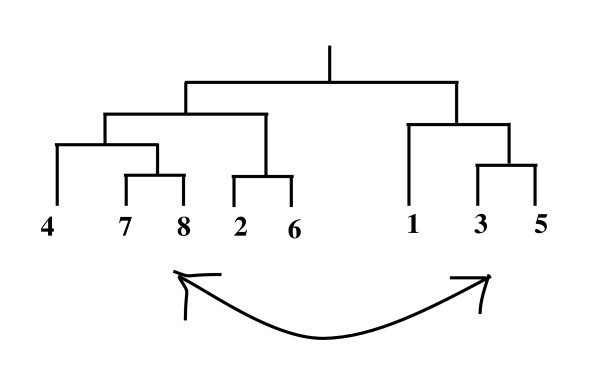
**Shuffling mechanism, sequence numbers 8 and 3 interchange their positions**.

In this case, two sequence numbers are selected randomly from one tree (either TR1 or TR2). Then, these two sequences exchange their positions to make a new tree. Figure 4 shows the function of Mechanism 2.

### Fitness

The Weighted Sum of Pair Method (WSPM) is commonly used as a fitness measure for MSAs. For it, each column in an alignment is scored by summing the product of the scores of each pair of symbols and their pair weight. The score of the entire alignment is then summed over all column scores by using equations (3) and (4).

(3) $$S=\overset{L}{\underset{l=1}{\sum }}S_{l}\;where\;,\;\;S_{l}=\overset{N-1}{\underset{i=1}{\sum }}\overset{N}{\underset{j=i+1}{\sum }}W_{ij}\text{cos}t\left(A_{i},A_{j}\right)$$

Here, *S *is the cost of the multiple alignments. *L *is the length (columns) of the alignment; *S*_*l *_is the cost of the *l*-th column of *L *length. *N *is the number of sequences, and *W*_*ij *_is the weight of sequence *i *and *j*. In CLUSTAL W, the weight is calculated for each sequence and the pair weight is the product of the two sequence weights. *cost(A*_*i*_*,A*_*j*_*) *is the alignment score between the two aligned sequences *A*_*i *_and *A*_*j*_. When *A*_*i *_≠ '-' and *A*_*j *_≠ '-' then *cost(A*_*i *_*,A*_*j*_*) *is determined either from the PAM (Percentage of Acceptable point Mutations) or BLOSUM [46] matrix. Also when *A*_*i *_= '-' and *A*_*j *_= '-' then *cost(A*_*i*_*,A*_*j*_*) = 0*. Finally, the cost function *cost(A*_*i *_*,A*_*j*_*) *includes the sum of the substitution costs of the insertion/deletions when *A*_*i *_≠ '-' and *A*_*j *_= '-' or *A*_*i *_= '-' and *A*_*j *_≠ '-', using a model with affine gap penalties as shown in (4).

(4) G=g+nx

Here, *G *is the gap penalty, *g *is the cost of opening a gap, *x *is the cost of extending the gap by one and *n *is the length of the gap.

In CLUSTAL W, the author used different weight matrices, which depend on the estimated divergence of the sequences to be aligned at each stage, and proposed dynamically changeable gap penalties to overcome the local minima issue. Therefore, in this research, the CLUSTAL W weighted scheme, the CLUSTAL W default gap penalties (gap opening penalty is -10 and the gap extension penalty is -0.20), and the PAM250 matrix, a mutation probability matrix, were considered for the WSPM fitness measure. Note that PAM250 is considered to be a good general matrix for protein database searching. Also, the PAM matrices have been developed based on global alignments. To calculate the weight of each sequence, we used the CLUSTAL W weight function.

To optimize the VDGA, we have used both the sum of pair and the weighted sum of pair methods for the fitness function in our research. However, we have only reported the weighted sum of pair scores as the algorithm with this method performed better than with the sum of pair fitness function. This is because of the features (such as selection and crossover) and the parameters used in our algorithm.

### Child Generation

For each individual in the initial population, the WSPM score is calculated, and the individuals are then sorted according to the descending order of their scores. To generate a child population of 100 individuals in any generation, the following three genetic operators are used.

I. Single point crossover.

II. Multiple point crossovers.

III. Mutation.

The following sub-section 'Selection of Parameters' considers the relative proportion of when these operators are used.

#### 1) Single Point Crossover

**Figure 5:**
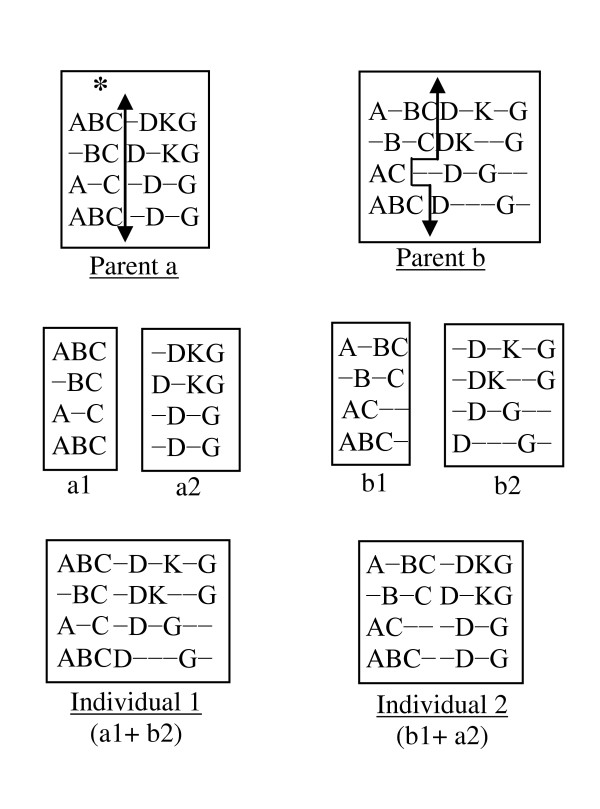
**Single point crossover**.

In this crossover, one individual is selected from the top 50% and another from the bottom 50% of the parent generation. The single point crossover [28] is implemented as shown in Figure 5. Its procedure is that first a column position is selected randomly as shown with a "*" in Figure 5. The parent having the better score is then divided vertically at that column. Let us assume that parent *a *has the better score column, so that this parent is separated vertically into two pieces. The second parent *b *is also divided into two pieces in such a way that each row of the first piece (and hence also the second piece) has the same number of elements as the first piece (and hence also the second piece) of the first parent. These pieces of these two parents are then exchanged and merged together to generate two new individuals as shown in Figure 5. However, only the better new individual is chosen to be a child.

#### 2) Multiple Point Crossovers

The parents' selection process is the same as for the single point crossover. For multiple point crossovers, each parent is divided into three pieces. These pieces are then exchanged between the parents and are then merged together to generate two new individuals. However, only the best one will be taken as a child. The crossover is implemented in two steps as described below.

#### Step 1

**Figure 6:**
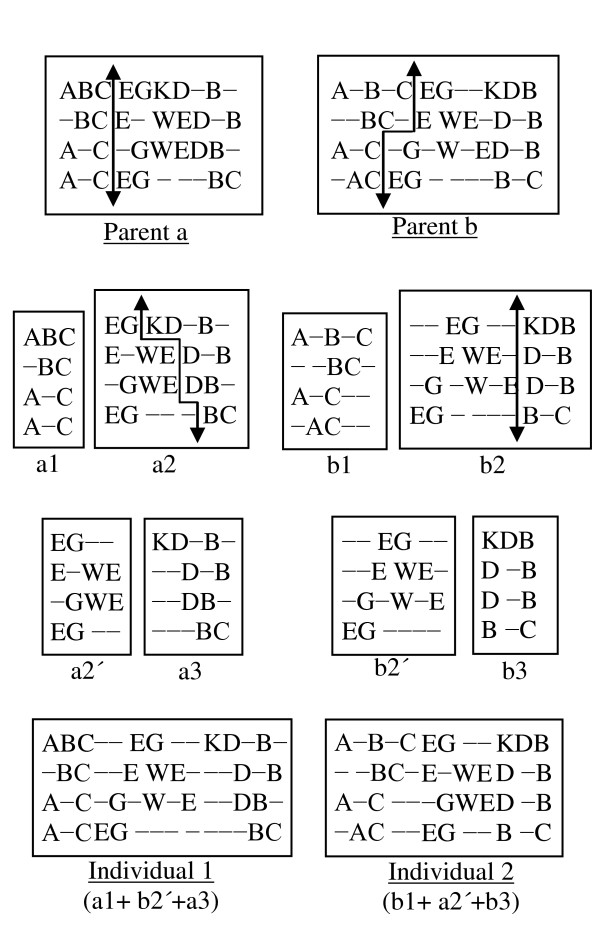
**Multiple point crossovers**.

To cut the first piece effectively, we compare the scores of the first 25% of columns for both parents. The parent having the better score is then divided vertically at that column. The other parent is also divided using the mechanism that was introduced in the single point crossover, as can be seen in Figure 6.

#### Step 2

We now have two pieces of each parent from Step 1. To create another piece, we follow the same procedure of Step 1, but considering the last 25% of columns (as shown in Figure 6). This gives us three pieces for each parent. To complete the crossover, the middle pieces are exchanged between the parents, and then all three pieces are merged together to generate the two new individuals as shown in Figure 6.

In Figure 6, the lengths (columns) of the two parents (parent *a *and parent *b*) are 10 and 12 respectively. The first 25% of the columns of the 1^st ^parent are two and a half (2.5) columns. In that case, we considered 3 columns. In the first step, parent *a *has the better score in the first 25% columns. Therefore, Parent *a *is divided first and Parent *b *is tailored according to Parent *a*. After the division, we have two pieces from each parent (*a*1 and *a*2 from Parent *a*; *b*1 and *b*2 from Parent *b*). In the 2^nd ^step, Parent *b *has the better score in the last 25% of columns. Therefore, Parent *b *is divided first and Parent *a *is tailored accordingly. This division provides two new pieces for each parent (*a*2' and *a*3 from *a*2; *b*2' and *b*3 from *b*2). Next, two new individuals are generated by connecting the pieces as (*a*1+*b*2'+*a*3) and (*b*1+*a*2'+*b*3). From these two individuals, the better one is selected as a child.

#### 3) Mutation

**Figure 7:**
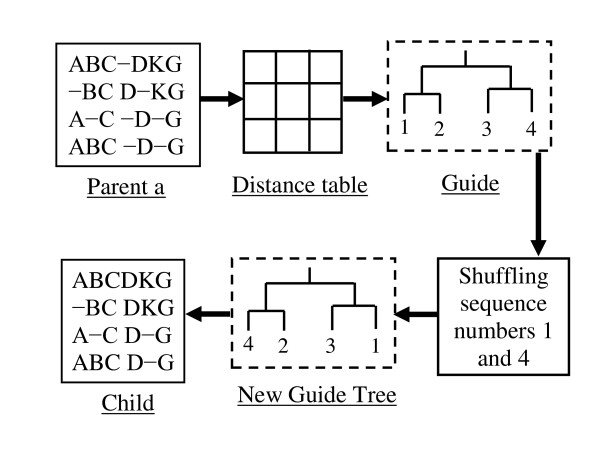
**Mutation**.

One individual (MSA) is randomly selected from the whole population. From this MSA, the distance among sequences are calculated and stored in a distance table. The new guide tree is constructed from this calculated distance. In the new guide tree, the sequence numbers are shuffled to find a better guide tree and the MSA of the new guide tree is considered as a mutated child. This process is repeated until 50 sequential unsuccessful attempts occur. If this operator finds a better guide tree then it is considered as a new child, otherwise there is no effect on this generation. The procedure of mutation is shown in Figure 7.

#### 4) Elitism

This common GA approach is used, whereby the best solution is passed on unchanged to the next generation.

### Vertical Division

**Figure 8:**
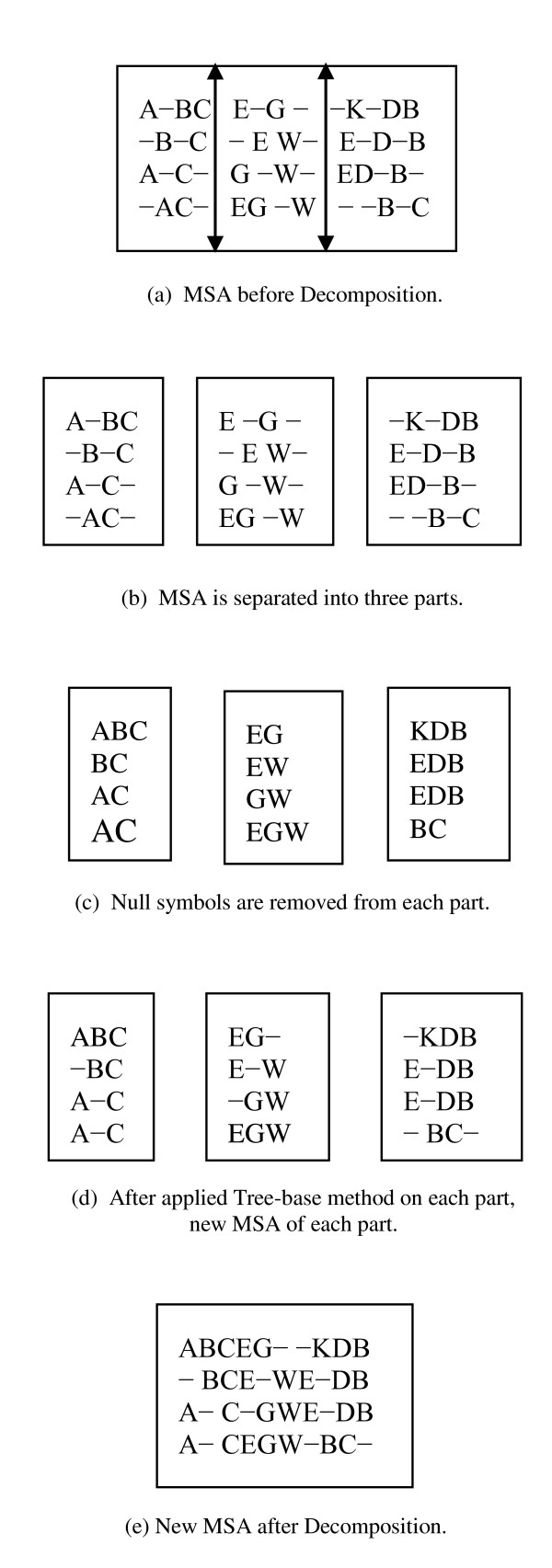
**Vertical Division Process**.

For Vertical Division, we first separated (decomposed) each alignment vertically into two or more sub-alignments as shown in Figure 8(a). After that, the null symbols "-" were removed from each decomposed part. Then the guide tree method was applied in each decomposed part. Therefore, we received a new alignment from each decomposed part, which may or may not be the same as the previous part. Now all of the new decomposed parts are connected together. In this way we obtain a new alignment. If the new alignment is better than the previous one, then the new alignment is kept, rather than the previous alignment. The working stages of this process are illustrated in Figure 8.

Figure 8(a) shows a multiple sequence alignment (MSA) before Vertical Division. To explain the process of Vertical Division, three divisions are considered as shown in Figure 8(b). Next, the null symbols are removed from each decomposed part as shown in Figure 8(c). Following that, the tree-base method is applied with each decomposed part and the new alignment of each decomposed part is determined as shown in Figure 8(d). Finally, the new alignments of the three decomposed parts are combined and thus we obtain the new alignment after the process of Vertical Division, as shown in Figure 8(e).

The Vertical Division step is applied in two places inside the proposed algorithm: once after the initial generation, and then after each child generation. We do this so we can apply the Vertical Division technique on aligned sequences. This is due to the fact that if the sequences are of different lengths, the Vertical decomposition of unaligned sequences may introduce either too many or too few residues in each decomposed part. Therefore, it could be difficult for the Vertical decomposition of an unaligned sequence to produce a good solution.

#### Motivation for Vertical Division

It is known that smaller sequences can be aligned quickly with a high level of accuracy [48]. This motivates us to divide the longer sequences into smaller parts, align them separately, and then combine them to generate aligned complete sequences. We have also observed in aligned sequences that sometimes the residue (character) is almost trivially incorrectly aligned. However, for larger sequence lengths and for a large number of sequences, it is computationally expensive to improve the multiple sequence alignments by simply shuffling the null symbols for the entire length of the MSA. Therefore, we have proposed an alternative, but efficient option to improve the MSA.

### New Generation

**Figure 9:**
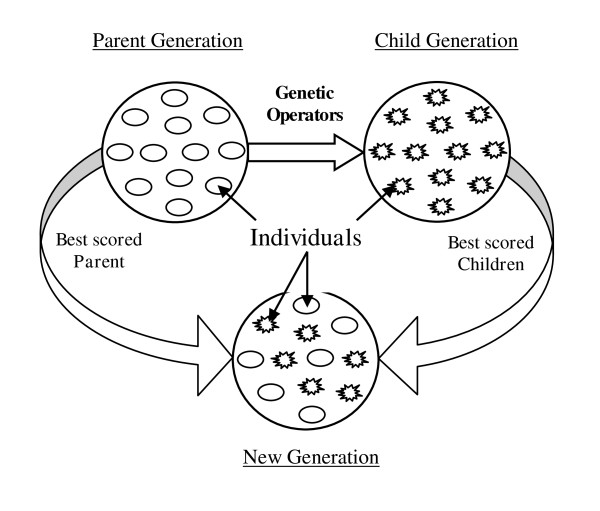
**Graphical presentation of generating New Generation**.

To form the new population, the best 50% of the combined parents and children are selected while ensuring that there is no duplication of the individuals. We must mention here that we have also studied other splits, such as 40-60 (parent-child) and 60-40. The study results showed that the 50-50 split outperforms the 40-60 and 60-40 splits with an average improvement of 4.38% and 7.66% respectively. Therefore, we have chosen the 50-50 mix with the proposed VDGA, which ensures a better balance between exploration and exploitation. The population size of 100 is chosen as it was used in SAGA [28]. Moreover, we have experimented with other population sizes which are discussed in a later section. The process of forming a new generation is demonstrated in Figure 9. The new population is then considered as the parent population in the next generation and so is used to continue the evolution process of VDGA.

### Termination Condition

The best solution in each generation is recorded. If the best solution remains the same in 100 consecutive generations, the algorithm will be terminated. We have set this termination condition based on our experimental observations. We have tested our VDGA algorithm for up to 300 generations after getting the best solution, and we have observed that the best solution was hardly changed and that the variation of the average solution per generation was also insignificant.

## Results and Discussion

### Test Datasets

In order to evaluate our proposed approach, we have solved a good number of test datasets from the benchmark BAliBase alignment database. The original BAliBase version 1.0 [49] consists of 142 reference alignments with over 1000 sequences. BAliBase version 2.0 [36] is an improved version, which was extended from version 1 to have 167 reference alignments and over 2100 sequences. BAliBase version 2.0 contains eight reference sets. Each reference set has a variety of alignment problems. Reference 1 contains small numbers of equidistant sequences. The orphan or unrelated sequences are considered in Reference 2. Reference 3 contains a pair of divergent subfamilies where the two groups are less than 25% identical. Reference 4 contains long terminal extensions, and Reference 5 contains large internal insertions and deletions. Lastly, references from 6 to 8 contain test case problems where the sequences are repeated and the domains are inverted. The details of the datasets used for the experiments are given in the 'Quality of Solution' subsection.

### Experimental Study

In this section, we have first analyzed the performance of the vertical decompositions with both the guide tree and the genetic algorithm. From that, we have proposed the appropriate number of decompositions with the proposed GA method. Finally, we have compared our algorithm (VDGA) with other well-known methods. In this research, we have analyzed our results based on 10 independent runs. In comparison, MSA-GA and RBT-GA used 5 and 10 runs respectively.

### Vertical Division/Decomposition with Guide-tree

Initially, multiple sequence alignments were done without a GA but solely by the application of the guide tree method followed by the results of that being decomposed vertically into two, three and four parts, namely Decomp_2, Decomp_3 and Decomp_4 respectively with the null symbols ("-") being removed from each part. Then we again applied the guide tree methods in each part of the decomposition. These alignments were evaluated by WSPM and their corresponding BAliscore were also calculated. Both of these scores are reported in Table 1. In this case, we carried out experiments for 34 datasets from references 2 and 3 of the BAliBase (version 2.0) [36] datasets. We also performed a non-parametric statistical test, namely the Wilcoxon Signed Rank Test [50], with respect to the WSPM score and also BAliscore as shown in Table 2.

**Table 1:** Performance of Decomposition techniques on the Solutions of the guide tree

**Reference No**.	Name of Dataset	Guide-tree		Decomp_2		Decomp_3		Decomp_4	
		
		WSPM	*Correspon-ding BAliscore*	WSPM	*Correspond-ing BAliscore*	WSPM	*Correspond-ing BAliscore*	WSPM	*Correspond-ing BAliscore*
Ref. 2	1aboA	-1001.96	*0.552*	-922.417	*0.566*	-954.859	*0.573*	-893.733	*0.587*
	
	1idy	17.78	*0.874*	17.78	*0.874*	128.604	*0.948*	57.494	*0.880*
	
	1csy	-394.56	*0.460*	-375.955	*0.568*	-293.088	*0.671*	-279.613	*0.747*
	
	1r69	-153.23	*0.716*	-122.574	*0.750*	-112.983	*0.780*	-109.973	*0.723*
	
	1tvxA	118.32	*0.916*	118.322	*0.936*	118.322	*0.936*	127.866	*0.941*
	
	1tgxA	-141.68	*0.798*	-28.893	*0.801*	-26.709	*0.815*	54.808	*0.809*
	
	1ubi	-245.02	*0.641*	-151.429	*0.618*	-213.452	*0.661*	-215.21	*0.671*
	
	1wit	-572.81	*0.673*	-547.512	*0.654*	-551.823	*0.670*	-456.085	*0.741*
	
	2trx	411.79	*0.944*	423.688	*0.948*	567.091	*0.945*	546.10	*0.977*
	
	1sbp	-226.99	*0.710*	-226.99	*0.710*	-200.282	*0.733*	-163.487	*0.710*
	
	1havA	-666.52	*0.783*	-499.625	*0.787*	-552.664	*0.787*	-470.297	*0.793*
	
	1uky	-368.69	*0.651*	-227.468	*0.634*	-320.261	*0.629*	-213.937	*0.710*
	
	2hsdA	-1139.99	*0.679*	-848.958	*0.790*	-1020.644	*0.729*	-1015.369	*0.721*
	
	2pia	-564.48	*0.811*	-466.756	*0.799*	-485.944	*0.801*	-341.627	*0.764*
	
	3grs	-649.94	*0.698*	-577.104	*0.714*	-609.335	*0.709*	-460.447	*0.746*
	
	Kinase	-1112.18	*0.763*	-973.132	*0.741*	-845.541	*0.771*	-924.093	*0.747*
	
	1ajsA	1433.11	*0.879*	1481.384	*0.901*	1504.135	*0.895*	1502.130	*0.881*
	
	1cpt	-1406.04	*0.732*	-1406.03	*0.732*	-1392.476	*0.725*	-1200.642	*0.751*
	
	1lvl	-2855.24	*0.705*	-2855.241	*0.705*	-2281.80	*0.726*	-2687.77	*0.749*
	
	1pamA	-1895.88	*0.699*	-1238.72	*0.750*	-716.222	*0.795*	-1311.726	*0.720*
	
	1ped	1556.41	*0.890*	1556.407	*0.902*	1610.442	*0.901*	1612.574	*0.907*
	
	2myr	664.52	*0.734*	753.00	*0.757*	775.25	*0.751*	760.204	*0.754*
	
	4enl	-42.30	*0.802*	12.135	*0.812*	116.309	*0.822*	66.895	*0.824*

Ref. 3	1idy	-1408.27	*0.236*	-1378.605	*0.303*	-1278.872	*0.256*	-1274.323	*0.447*
	
	1r69	-1735.897	*0.541*	-1662.407	*0.551*	-1735.897	*0.562*	-1735.897	*0.567*
	
	1ubi	-1417.25	*0.308*	-1381.00	*0.264*	-1271.455	*0.406*	-1396.996	*0.321*
	
	1wit	-1059.74	*0.553*	-849.307	*0.628*	-1015.695	*0.644*	-806.003	*0.676*
	
	1uky	-5417.19	*0.364*	-4981.878	*0.387*	-5283.305	*0.415*	-5417.186	*0.364*
	
	kinase	-1112.18	*0.736*	-973.132	*0.741*	-845.541	*0.771*	-924.093	*0.747*
	
	1ajsA	-4597.06	*0.264*	-4597.06	*0.264*	-4551.029	*0.264*	-4597.06	*0.264*
	
	1pamA	-4217.03	*0.680*	-3997.312	*0.687*	-2981.082	*0.746*	-4107.983	*0.694*
	
	1ped	-2296.46	*0.574*	-1789.276	*0.577*	-1512.652	*0.682*	-1832.71	*0.589*
	
	2myr	-8545.30	*0.318*	-8516.803	*0.359*	-8163.882	*0.355*	-7483.46	*0.347*
	
	4enl	-783.58	*0.600*	-582.216	*0.607*	-216.91	*0.635*	-470.220	*0.618*

**Table 2:** Statistics test of Table 1 with Wilcoxon Signed Rank Test (based on negative ranks)

	w.r.to WSPM			w.r.to Corresponding BAliscore		
	
	Decomp2-Guide tree	Decomp3-Guide tree	Decomp4-Guide tree	Decomp2-Guide tree	Decomp3-Guide tree	Decomp4-Guide tree
'+' ve Rank	29	33	32	23	29	29

'-' ve Rank	2	0	0	6	4	2

Ties	3	1	2	5	1	3

Z	-4.782	-5.012	-4.937	-2.520	-4.460	-4.174

Asymp. Sig. (2-tailed)	0.000	0.000	0.000	0.012	0.000	0.000

Comparing with the WSPM solutions of the guide tree, it is observed from Table 1 and Table 2 that Decomp_2 was better in 29 test cases and found the same best results in 3 test cases out of 34 test cases. Decomp_3 found the same best results in 1 test case and successfully found better solutions in 33 test cases. Moreover, Decomp_4 was better in 32 test cases and in two test cases it found the same best results. The non-parametric test shows that these vertical decompositions are significantly better than the guide tree.

On the other hand, comparing with the corresponding BAliscore solutions of the guide tree, Decomp_2 was better in 23 test cases and found the same best results in 5 test cases, Decomp_3 was better in 29 test cases and found the same bestn results in one test case, Decomp_4 was also better in 29 test cases and in three test cases it found the same best results out of 34 test cases. The non-parametric test also shows the significant advantage of these vertical decompositions with guide tree.

From the experimental observation it is clear that the multiple sequence alignments using vertical decompositions perform better than that of the guide tree method in most of the test cases. In a very few cases, these techniques perform badly, or the same as, the guide tree. Although in general Decomp_3 performed best, sometimes the other decompositions performed better in some cases. After these experimental and statistical analyses with respect to the objective function and benchmark scores, we can safely conclude that the vertical decompositions can play an important role in improving existing solutions, and there is a possibility to find a better multiple sequence alignment if the vertical decompositions are used with an evolutionary approach (such as a genetic algorithm).

### Analysis of Vertical Decomposition with GA

In this section, we have first analyzed the selection of parameters for VDGA and then discussed the appropriate number of decompositions. In some of the following analysis, the decomposition was kept inactive to judge the parameter's effect individually.

#### • Selection of Parameters

In the proposed VDGA algorithm, we have used two basic search operators: crossover and mutation. In order to determine the probabilities of crossover and mutation, we have excluded the decompositions from GA and have carried out five different experiments (100% crossover; 60% crossover & 40% mutation; 50% crossover & 50% mutation; 40% crossover & 60% mutation; and 100% crossover), using ten randomly selected BaliBase datasets (version 2.0) [36]. Our GA with the 50%-crossover & 50%-mutation option obtained the best solutions for seven out of ten datasets, the 60%-crossover & 40%-mutation for two and 40%-crossover & 60%-mutation for one (but as the same as that of 50%-crossover & 50%-mutation). The options 100%-crossover achieved the best solution in one test case. However, the option 100%-mutation did not achieve any best solution. The solutions obtained by the 50%-crossover & 50%-mutation for the other three datasets were close to the best scores. The GA with 50% crossover & 50% mutation (which is the 3^rd ^mix) achieved an average improvement of 4.66% over the first mix, 0.421% over the second mix, 1.02% over the fourth mix and 4.93% over the fifth mix. From the experimental performance, we have decided to use 50% probabilities of crossover and mutation with VDGA. For the 50% crossovers, single point crossover is selected for half of them and double point crossover is used for the other half. These selections were also decided based on experimental analysis.

To form the child population from the parent population, we have divided the population into two groups based on their fitness values. Then one individual is selected from the top 50% and another from the bottom 50% for crossover. However, the new population is formed by taking the best individuals from the combined previous parents and children.

We have chosen the population size of 100 as used in SAGA [28]. However, we have run experiments using the population sizes 50, 100 and 200 with 50% crossover & 50% mutation. The performance of VDGA with a population size 100 is significantly better than that of with a population size 50 and shows no significant difference with a population size 200.

#### • Effect of Operators and Initial Population

The proposed genetic algorithm, VDGA, uses an improved initial population and new genetic operators that contribute to it performing better than other algorithms. To analyze the effect of these two components on the algorithm's performance, we have excluded the decomposition part from our algorithm and tested two sets of new experiments. In the first set, GA was run with a *randomly generated *initial population (instead of our improved initial population), and the second set used a hill climbing approach (for searching instead of our GA) starting from the improved initial population. WSPM was used as the fitness measure. Based on the corresponding BAliscore of the best found WSPM solution, the full algorithm achieved an average improvement of 8.43% compared to the same with the randomly generated initial population and 12.79% compared to the hill climbing approach. The first set of experiments thus proved the superiority of our proposed initial population, and the second set demonstrated the strength of our proposed genetic search operators.

#### • Staging of Decomposition with VDGA

To determine the appropriate stage for using decomposition inside VDGA, we have carried out experiments on eight datasets from BAliBase. We have tested our algorithm using decompositions in three cases. In the first case, the decompositions were used only after the initial generation, in the second case only after each child generation and finally after all generations. For Decomp_2, the VDGA with the third case improved the average solution by 1.74% better than the first case and 0.6% better than the second case. For Decomp_3, the VDGA with the third case improved the average solution of 2.91% compared to the first case and 0.87% compared to the second case. Moreover, Decomp_4 with the third case improved the average solution by 3.08% more than the first case but the second case performed better than the third case. Although VDGA with the third case for decomposition 4, did not perform better than with the second case, with other decompositions (Decomp_2 and Decomp_3) VDGA achieved much better average performance when the decompositions were used after all (initial and each child) generations, in comparison to the first and second cases. From these experimental observations, we have decided to use the decomposition technique after all generations.

#### • Performance of Decompositions with GA

In order to judge the performance of the Vertical Division with GA, we have also performed experiments for 34 datasets from BAliBase 2.0, where we have considered the GA and also the VDGA (Vertical Decompositions with GA) algorithms. The algorithms were each executed for 10 independent runs. For each dataset, the best and average WSPM scores out of the 10 runs were recorded, and the corresponding BAliscore of the best found WSPM solution was also calculated. The best and average WSPM scores and the corresponding BAliscore are reported in Table 3. To compare with other results and algorithms, we have carried out non-parametric statistical testing with the Wilcoxon Signed Rank test with respect to the best WSPM score, the average score and also with the corresponding BAliscore as shown in Table 4.

**Table 3:** Performance of Decomposition techniques with Genetic Algorithm

		VDGA Without Decomposition			VDGA Decomp_2			VDGA Decomp_3			VDGA Decomp_4		
	
**Reference No**.	Name of Dataset	Best WSPM	Ave. WSPM	*Corresponding BAliscore*	Best WSPM	Ave. WSPM	*Corresponding BAliscore*	Best WSPM	Ave. WSPM	*Corresponding BAliscore*	Best WSPM	Ave. WSPM	*Corresponding BAliscore*
Ref. 2	1aboA	-377.456	-454.63	*0.796*	-301.74	-355.41	*0.723*	-234.25	-278.37	*0.791*	-334.12	-448.12	*0.679*
	
	1idy	408.98	380.81	*0.989*	489.85	480.74	*0.981*	516.31	498.62	*0.992*	430.70	303.702	*0.992*
	
	1csy	-69.93	-101.09	*0.764*	-62.52	-66.29	*0.731*	-51.203	-59.42	*0.885*	-71.592	-86.019	*0.831*
	
	1r69	-28.937	-50.94	*0.965*	-1.453	-5.086	*0.859*	18.728	10.32	*0.934*	33.625	22.863	*0.874*
	
	1tvxA	334.38	315.46	*0.920*	364.01	321.49	*0.944*	387.32	384.51	*0.974*	384.79	286.39	*0.944*
	
	1tgxA	342.38	307.97	*0.878*	339.61	321.15	*0.867*	367.42	342.23	*0.878*	390.56	376.289	*0.850*
	
	1ubi	36.30	4.93	*0.767*	42.1	6.782	*0.732*	48.14	22.39	*0.778*	45.71	39.649	*0.794*
	
	1wit	-120.21	-183.60	*0.851*	-108.91	-199.764	*0.875*	-102.45	-113.59	*0.815*	-115.34	-186.919	*0.774*
	
	2trx	903.23	855.29	*0.986*	929.39	905.042	*0.959*	957.92	945.45	*0.986*	944.13	921.391	*0.986*
	
	1sbp	-19.79	-75.19	*0.765*	-12.58	-18.98	*0.782*	-34.26	45.66	*0.772*	-29.019	-34.766	*0.778*
	
	1havA	49.62	33.57	*0.879*	189.46	213.42	*0.884*	227.25	147.09	*0.846*	240.39	210.34	*0.884*
	
	1uky	-84.92	-121.98	*0.808*	21.91	-9.297	*0.845*	69.57	42.91	*0.891*	-76.23	-106.861	*0.872*
	
	2hsdA	-389.79	-443.99	*0.796*	-365.25	-426.28	*0.856*	-309.64	-355.36	*0.829*	-334.22	-391.32	*0.742*
	
	2pia	-146.38	-223.91	*0.826*	-125.35	-143.025	*0.847*	-53.76	-75.84	*0.850*	-33.85	-48.513	*0.839*
	
	3grs	-142.16	-210.66	*0.746*	-118.195	-142.41	*0.717*	-136.70	-164.49	*0.751*	-69.65	-131.917	*0.781*
	
	Kinase	-191.16	-224.46	*0.799*	-129.92	-263.61	*0.825*	-180.11	-195.82	*0.888*	-145.51	-221.963	*0.812*
	
	1ajsA	1956.94	1920.89	*0.899*	1938.87	1918.284	*0.906*	2011.39	1958.0	*0.905*	1900.35	1827.312	*0.902*
	
	1cpt	-435.69	-490.22	*0.875*	-402.59	-551.276	*0.869*	-325.41	-413.98	*0.812*	-410.893	-504.988	*0.853*
	
	1lvl	-826.15	-916.93	*0.781*	-720.022	-884.03	*0.803*	-688.34	-810.04	*0.819*	-772.43	-1003.08	*0.816*
	
	1pamA	-974.64	-1019.11	*0.814*	-986.278	-1040.69	*0.857*	-941.56	-1012.69	*0.863*	-939.83	-1045.98	*0.853*
	
	1ped	1940.82	1862.50	*0.912*	1911.36	18670.39	*0.935*	2003.19	1996.74	*0.947*	1998.13	1979.245	*0.943*
	
	2myr	13970.32	13865.22	*0.822*	13991.45	13292.104	*0.806*	14123.57	14115.91	*0.830*	14095.12	14042.641	*0.808*
	
	4enl	1386.81	1299.04	*0.896*	1161.56	1107.331	*0.890*	1329.23	1248.89	*0.889*	920.982	895.165	*0.899*

Ref. 3	1idy	-512.34	-588.37	*0.601*	-588.25	-660.92	*0.446*	-496.91	-554.09	*0.599*	-482.98	-518.89	*0.569*
	
	1r69	-1103.34	-1174.5	*0.709*	-1099.76	-1181.71	*0.724*	-1081.08	-1124.14	*0.733*	-1056.54	-1083.03	*0.765*
	
	1ubi	-959.62	-1004.26	*0.386*	-948.10	-1018.84	*0.398*	-871.85	-960.94	*0.414*	-916.78	-1005.13	*0.410*
	
	1wit	-223.56	-263.99	*0.758*	-319.695	-332.971	*0.833*	-241.51	-309.58	*0.873*	-233.687	-273.302	*0.867*
	
	1uky	-3565.98	-3662.99	*0.468*	-3412.58	-3595.6	*0.469*	-3181.7	-3308.02	*0.481*	-3287.773	-3328.27	*0.526*
	
	kinase	-294.62	-357.70	*0.828*	-225.67	-276.98	*0.870*	-153.90	-213.56	*0.890*	-193.57	-248.795	*0.887*
	
	1ajsA	-4199.49	-4250.98	*0.311*	-3375.033	-3467.24	*0.383*	-3136.73	-3463.15	*0.453*	-3339.93	-3439.16	*0.408*
	
	1pamA	-2106.98	-2163.54	*0.835*	-1989.045	-2179.37	*0.853*	-1843.03	-2015.55	*0.788*	-1889.85	-1903.52	*0.792*
	
	1ped	-1199.41	-1251.38	*0.813*	-683.44	-811.29	*0.848*	-411.75	-457.17	*0.893*	-721.65	-801.563	*0.783*
	
	2myr	-6498.07	-6574.31	*0.513*	-6180.015	-6305.04	*0.586*	-5523.14	-5801.53	*0.651*	-5962.83	-6103.28	*0.519*
	
	4enl	-45.86	-72.34	*0.800*	98.51	19.426	*0.836*	162.46	121.04	*0.866*	102.08	80.624	*0.866*

**Table 4:** Statistics test of Table 3 with Wilcoxon Signed Ranks Test

Based on Best WSPM score						
	**VDGA(Decomp2-without_Decomp.)**	**VDGA(Decomp3-without_Decomp.)**	**VDGA(Decomp4-without_Decomp.)**	**VDGA(Decomp3-Decomp2)**	**VDGA(Decomp4-Decomp2)**	**VDGA(Decomp4-Decomp3)**

'+' ve Rank	27	31	29	**31**	22	**11**

'-' ve Rank	7	3	5	**3**	12	**23**

Ties	0	0	0	**0**	0	**0**

Z	-3.189^a^	-4.625^a^	-4.026^a^	-4.625^a^	-1.975^a^	-2.983^b^

Asymp. Sig. (2-tailed)	0.001	0.000	0.000	**0.000**	0.048	**0.003**

Based on Avg. WSPM score						

	VDGA(Decomp2-without_Decomp.)	VDGA(Decomp3-without_Decomp.)	VDGA(Decomp4-without_Decomp.)	VDGA(Decomp3-Decomp2)	VDGA(Decomp4-Decomp2)	VDGA(Decomp4-Decomp3)

'+' ve Rank	22	32	24	**31**	22	**11**

'-' ve Rank	12	2	10	**3**	12	**23**

Ties	0	0	0	**0**	0	**0**

Z	-2.197^a^	-4.744^a^	-2.949^a^	-4.078^a^	-1.342^a^	-2.829^b^

Asymp. Sig. (2-tailed)	0.028	0.000	0.003	**0.000**	0.180	**0.005**

Based on Corresponding BAliscore						

	VDGA(Decomp2-without_Decomp.)	VDGA(Decomp3-without_Decomp.)	VDGA(Decomp4-without_Decomp.)	VDGA(Decomp3-Decomp2)	VDGA(Decomp4-Decomp2)	VDGA(Decomp4-Decomp3)

'+' ve Rank	22	24	23	**26**	19	**9**

'-' ve Rank	12	8	10	**8**	13	**22**

Ties	0	2	1	**0**	2	**3**

Z	-1.496^a^	-3.020^a^	-1.439^a^	-2.967^a^	-0.926^a^	-2.215^b^

Asymp. Sig. (2-tailed)	0.135	0.003	0.150	**0.003**	0.355	**0.027**

a. Based on negative ranks.

b. Based on positive ranks.

Comparing with GA in terms of the best WSPM score, VDGA with Decomp_2 was better in 27, and worse in 7 test cases as shown in Table 3 and Table 4. The *Z *value (-3.189) and the significant test result (P = 0.001 < = 0.05) in Table 4 show that VDGA with Decomp_2 is significantly better than GA. VDGA with Decomp_3 found better solution in 31 test cases, and the statistical test results (Z=-4.625; P = 0.00) prove that the VDGA with Decomp_3 is also significantly better than GA. Moreover, VDGA with Decomp_4 is also significantly better than GA. In doing so it found better solutions in 29 test cases and worse in 5 test cases. Among these three decompositions, VDGA with Decomp_3 successfully found better MSAs in a maximum of 31 test cases out of 34.

When considering the average WSPM score, the VDGA with all three decompositions (Decomp_2, Decomp_3 and Decomp_4) are significantly better than GA. In doing so VDGA with Decomp_2 was better in 22 test cases, Decomp_3 was better in 32 and Decomp_4 was in 24. In this comparison, VDGA with Decomp_3 successfully found better solutions in a maximum number of test cases in comparison to the other two decompositions. The statistical test results in Table 4 show that VDGA with Decomp_2 is not significantly different than VDGA with Decomp_4.

Also comparing with the corresponding BAliscore of the best finding WSPM solution, VDGA with Decomp_2 was better in 22 test cases, Decomp_3 was in 24 and Decom_4 was in 23. The significance test in Table 4 shows that VDGA with Decomp_2 and also with Decomp_4 are not significantly different than GA. However, VDGA with Decomp_3 is significantly better than GA. Moreover, VDGA with Decomp_2 and with Decomp_4 are not significantly different. VDGA with Decomp_3 is significantly better than the two other decompositions.

From the above experimental results and their statistical analyses, it is clear that VDGA with Decomp_3 found better solutions in more test cases than the other two decompositions and GA, and it is significantly better than GA and VDGA with the other two decompositions with respect to the best and average WSPM score and the corresponding BAliscore. As we have considered test sequences with length of up to 1000 in this research, we considered that the appropriate number of divisions for VDGA is 3 (Decomp_3) for sequences up to 1000 length. Further experiments would be required to determine the best setting for longer sequences.

#### • Computational Effort and Convergence

The computational time required for finding good multiple sequence alignments is dependent on the sequence length, the number of sequences, and the similarities of the sequences. In addition, the choice of algorithmic parameters also plays an important role. We have tried to develop a relationship between the computational time required (with our algorithm) and the sequence length and sequence numbers. However, it is hard to make any firm conclusion based on linear/nonlinear regression analysis.

**Figure 10:**
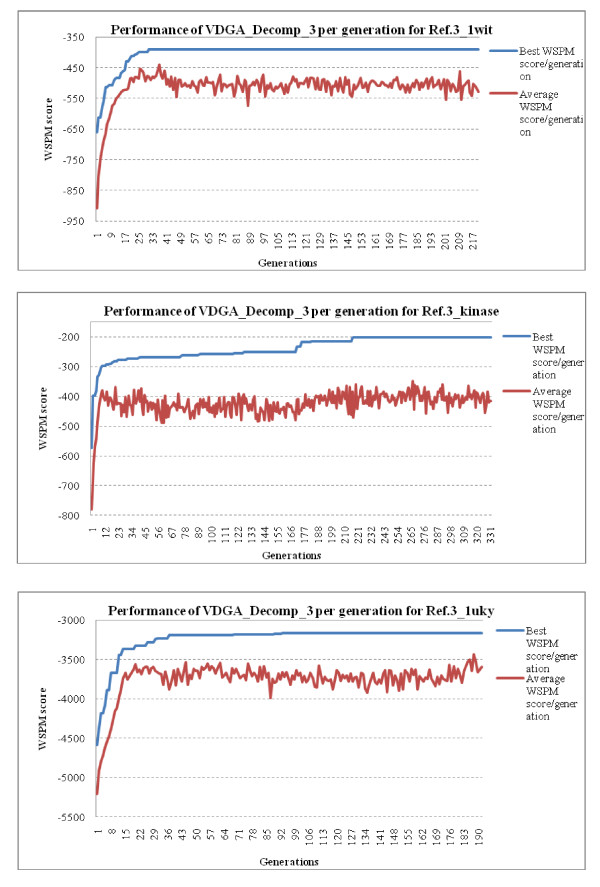
**Graphical presentations of the performance of the VDGA_Decomp_3 method w.r.to the Best and Average WSPM score per generation**.

To show the convergence behavior of our algorithm (VDGA with Decomp_3), we have plotted the best and the average WSPM scores against the number of generations. As examples, three such plots (for one specific run) for three datasets from reference 3 are presented in Figure 10. These graphs show that our algorithm improved both the best and the average scores very rapidly at the initial stage of the search process and that the best score then converged to a solution. This is the type of pattern we expect from good search algorithms. As of the plots, although the average scores do not converge, the rate of improvement for the best score in the later generations of the algorithm is insignificant.

### Quality of Solutions

To judge the quality of the solutions produced by our algorithm, we have considered only those benchmark datasets and algorithms that were considered in the papers reporting MSA-GA and RBT-GA, and that used BAliscore (an open source program of the BAliBase benchmark) to measure the accuracy of the solutions. The authors of MSA-GA considered the best solution of five runs for each dataset and reported the BAliscore. Moreover, the authors of RBT-GA also reported the best solution of ten runs with BAliscore. In our algorithm, we considered ten independent runs of each dataset and have used the corresponding BAliscore of the best found WSPM solution. BAliscore scores a solution (multiple sequence alignment) between 0.0 and 1.0. If the solution is identical with the corresponding manually created reference alignment then the score is 1.0. If nothing matches with the reference alignment then the score is 0.0. However, if some parts match with the reference alignment, then the score is in between 0.0 and 1.0.

In MSA-GA, the authors considered 28 test datasets from references 1 to 5 and reference 8. Among them, 18 datasets were from reference 1 and two were from each of the other reference datasets. However, currently BAliscore does not work for reference set 8. This is because of insufficient information supplied either by the reference alignment file or by the annotation file. Therefore, we excluded the two datasets of reference 8, thus leaving 26 for comparison. In RBT-GA, the author considered all 23 test datasets of reference 2, and 11 out of 12 from reference 3. In total, we considered 56 test datasets, including 18 from reference 1, 23 (all) from reference 2, 11 from reference 3, and 2 from each of references 4 and 5. All these datasets belong to the BAliBase 2.0 benchmark datasets.

### Problem Solving with VDGA_Decomp_3

For each of the 56 datasets, we have executed our algorithm for 10 independent runs and recorded the best, worst, and average WSPM scores with standard deviation, and the corresponding BAliscore of the best WSPM score in Table 5. The WSPM scores could be either positive or negative, as it depends on the level of similarity among the residues in the sequences. This is because, if the residues among the comparable sequences are similar, or partially similar, it needs a small number of null ('-') symbols to make an alignment of the sequences. In this case, the WSPM score of this alignment is positive. On the other hand, if the dissimilar parts among the sequences are high, a large number of null symbols are added to the alignment. In this case, the WSPM score becomes negative because of gap penalties. Note that high positive values and low negative values are considered as good scores. We must also mention here that the average scores in the 10 runs were not very different and hence the standard deviations were small.

**Table 5:** The Summery of the test results of VDGA_Decomp_3 method

Name of Experiment		Sequence Number	Sequence Length	With WSPM			
				
				Best Score	Avg. Score	Std	Corresponding BAliscore
Ref. 1	1idy	5	58	69.45	53.21	4.56	0.573
	
	1tvxA	4	69	34.98	21.32	7.91	0.267
	
	1uky	4	220	9.039	-25.48	19.30	0.449
	
	Kinase	5	276	-90.18	-91.90	1.46	0.545
	
	1ped	3	374	41.21	25.91	8.98	0.482
	
	2myr	4	474	-80.324	-82.725	3.26	0.359
	
	1ycc	4	116	-18.313	-32.67	9.21	0.755
	
	3cyr	4	109	21.6	14.56	4.85	0.821
	
	1ad2	4	213	59.61	43.67	6.98	0.941
	
	1ldg	4	675	89.21	51.81	7.91	0.906
	
	1fieA	5	442	236.42	235.84	0.47	0.930
	
	1sesA	5	63	283.20	275.60	5.41	0.962
	
	1krn	4	82	70.21	62.45	1.34	0.960
	
	2fxb	5	63	141.78	141.78	0	0.978
	
	1amk	5	258	105.89	78.31	12.21	0.984
	
	1ar5A	4	203	61.34	34.56	6.78	0.938
	
	1gpb	5	828	847.84	846.94	0.94	0.984
	
	1taq	5	928	612.66	608.22	5.10	0.959

Ref. 2	1aboA	15	80	-234.25	-278.37	35.77	0.691
	
	1idy	19	60	516.31	498.62	11.32	0.992
	
	1csy	19	99	-51.203	-59.42	6.61	0.885
	
	1r69	20	76	18.728	10.32	8.52	0.834
	
	1tvxA	16	69	387.32	384.51	1.69	0.974
	
	1tgxA	19	71	367.42	342.23	27.92	0.878
	
	1ubi	19	60	48.14	22.39	18.85	0.778
	
	1wit	20	106	-102.45	-113.59	8.83	0.815
	
	2trx	19	94	957.92	945.45	8.01	0.986
	
	1sbp	16	262	-34.26	45.66	8.49	0.772
	
	1havA	16	242	227.25	147.09	52.73	0.846
	
	1uky	23	225	69.57	42.91	20.69	0.891
	
	2hsdA	20	255	-309.64	-355.36	32.31	0.829
	
	2pia	16	294	-53.76	-75.84	14.46	0.850
	
	3grs	15	237	-136.70	-164.49	25.07	0.751
	
	Kinase	18	287	-180.11	-195.82	18.76	0.888
	
	1ajsA	18	389	2011.39	1958.0	39.43	0.905
	
	1cpt	15	434	-325.41	-413.98	68.15	0.812
	
	1lvl	23	473	-688.34	-810.04	73.21	0.819
	
	1pamA	18	511	-941.56	-1012.69	69.29	0.863
	
	1ped	18	388	2003.19	1996.74	6.95	0.947
	
	2myr	17	482	14123.57	14115.91	20.69	0.830
	
	4enl	17	440	1329.23	1248.89	48.67	0.889

Ref. 3	1idy	27	60	-496.91	-554.09	35.87	0.599
	
	1r69	23	78	-1081.08	-1124.14	29.22	0.733
	
	1ubi	22	97	-871.85	-960.94	58.08	0.414
	
	1wit	19	102	-241.51	-309.58	38.91	0.873
	
	1uky	24	220	-3181.7	-3308.02	113.83	0.481
	
	kinase	18	287	-153.90	-213.56	33.55	0.890
	
	1ajsA	28	396	-3136.73	-3463.15	203.97	0.453
	
	1pamA	19	511	-1843.03	-2015.55	127.14	0.788
	
	1ped	21	388	-411.75	-457.17	48.81	0.893
	
	2myr	21	482	-5523.14	-5801.53	305.24	0.651
	
	4enl	19	427	162.46	121.04	37.29	0.866

Ref. 4	1dynA	6	848	-101073.19	-101151.87	41.21	0.033
	
	Kinase2	7	468	-23090.7	-23051	23.12	0.542

Ref. 5	2cba	8	328	-755.07	-781.91	24.65	0.835
	
	S51	15	301	-1657.26	1821.16	103.23	0.743

For comparisons with other methods, we have taken from the published literature [34-36,51] the benchmark BAliscore results of those methods. The authors Gondro and Kinghorn (2007) in MSA-GA [34], Taheri and Zomaya, (2009) in RBT-GA [35], and Bahr et al. (2000) in BAliBase [36] reported only SPS scores for comparisons. Therefore, for them we have compared only the SPS scores. However, we also recorded the CS score of the proposed VDGA method from the BAliscore program. The comparisons are discussed below.

### Comparing VDGA with MSA-GA and Other Methods

**Figure 11:**
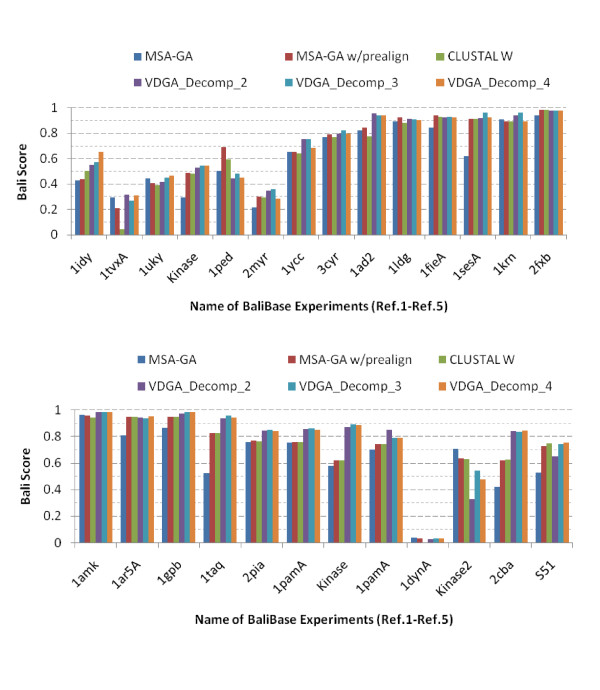
**Graphical presentations of the experimental results on MSA-GA selected datasets**.

To compare with the other methods, we have considered all three decompositions (Decomp_2, Decomp_3 and Decomp_4) with VDGA. The authors of MSA-GA [33] selected 28 test cases from references 1 to reference 5 and reference 8. As discussed earlier, we have considered 26 out of these 28 test cases. The results are provided in Table 6 and are plotted in Figure 11.

**Table 6:** Experiments on Selected Datasets of MSA-GA

**Reference No**.	Name of Dataset	MSA-GA	MSA-GA w/prealign	ClUSTAL W	VDGA_ Decomp_2	VDGA_Decomp_3	VDGA_Decomp_4
Ref. 1	1idy	0.427	0.438	0.500	0.550	0.651	**0.654**
	
	1tvxA	0.295	0.209	0.042	**0.316**	0.316	0.310
	
	1uky	0.443	0.405	0.392	0.416	0.459	**0.464**
	
	Kinase	0.295	0.488	0.479	0.531	0.545	**0.548**
	
	1ped	0.501	**0.687**	0.592	0.443	0.482	0.451
	
	2myr	0.212	0.302	0.296	0.347	**0.359**	0.282
	
	1ycc	0.650	0.653	0.643	0.752	**0.839**	0.685
	
	3cyr	0.772	0.789	0.767	0.797	**0.898**	0.797
	
	1ad2	0.821	0.845	0.773	**0.959**	0.950	0.941
	
	1ldg	0.895	**0.922**	0.880	0.914	0.946	0.903
	
	1fieA	0.843	**0.942**	0.932	0.926	0.960	0.927
	
	1sesA	0.620	0.913	0.913	0.917	**0.962**	0.923
	
	1krn	0.908	0.895	0.895	0.942	**0.960**	0.892
	
	2fxb	0.941	**0.985**	**0.985**	0.978	0.978	0.978
	
	1amk	0.965	0.959	0.945	0.982	**0.984**	0.982
	
	1ar5A	0.812	0.946	0.946	0.942	0.968	**0.954**
	
	1gpb	0.868	0.948	0.947	0.976	**0.984**	0.983
	
	1taq	0.525	0.826	0.826	0.938	**0.959**	0.944

Ref. 2	2pia	0.761	0.768	0.766	0.847	**0.850**	0.839
	
	1pamA	0.755	0.758	0.757	0.857	**0.863**	0.853

Ref. 3	Kinase	0.58	0.619	0.619	0.870	**0.890**	0.887
	
	1pamA	0.703	0.744	0.743	**0.853**	0.788	0.792

Ref. 4	1dynA	**0.038**	0.034	0.000	0.029	0.033	0.031
	
	Kinase2	**0.71**	0.635	0.630	0.330	0.542	0.478

Ref. 5	2cba	0.422	0.621	0.628	0.839	0.835	**0.846**
	
	S51	0.528	0.73	0.75	0.650	0.743	**0.756**

Average Score		0.627	0.695	0.679	0.727	**0.744**	0.735

In Table 6, the bold face data represents the best performing scores among the methods. From Table 6 and Figure 11, it is observed that VDGA with three decompositions achieved more accurate solutions than the others, in 19 out of 26 test cases, while Decomp_2 was better in three, Decomp_3 was in eleven and Decomp_4 was in six test cases. MSA-GA achieved better MSAs for only two test cases, MSA-GA w/prealign for four, and CLUSTAL W for one test case. However, both MSA-GA w/prealign and CLUSTAL W found the same solution in one test case. In seven test cases where VDGA did not achieve the best solutions, the solutions are close to the best solutions reported in the table. The VDGA with Decomp_3 achieved accurate results in the maximum number of test cases reported in this table.

**Figure 12:**
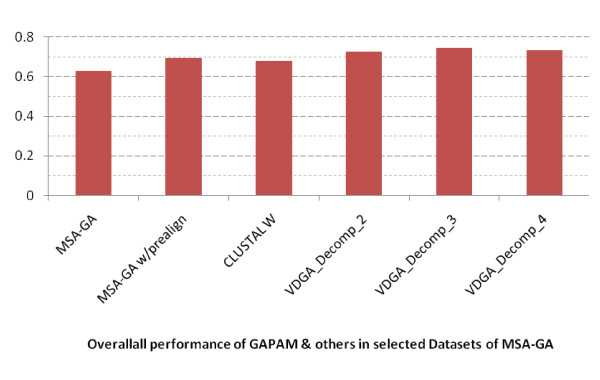
**Overall Performance of all methods in MSA-GA selected datasets**.

Based on the average scores reported in the bottom row in Table 6 and plotted in Figure 12, the VDGAs with decomposition (Decomp_2, Decomp_3 and Decomp_4) achieved higher scores and among these VDGA_Decomp_3 was the best for the 26 datasets. From the experimental results, we can claim that VDGA had better performance on these 26 test cases. The average CS score of VDGA for the MSA-GA selected datasets was 0.663.

### Comparing VDGA with RBT-GA

**Figure 13:**
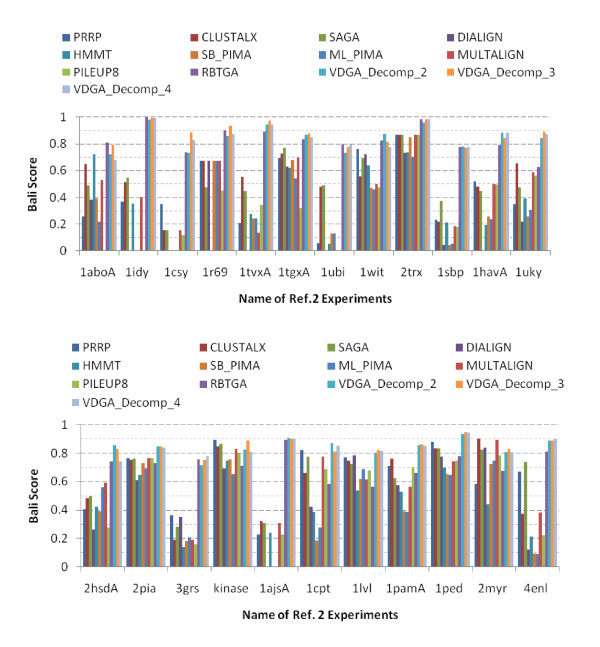
**Graphical presentations of the experimental results on reference 2 datasets**.

**Figure 14:**
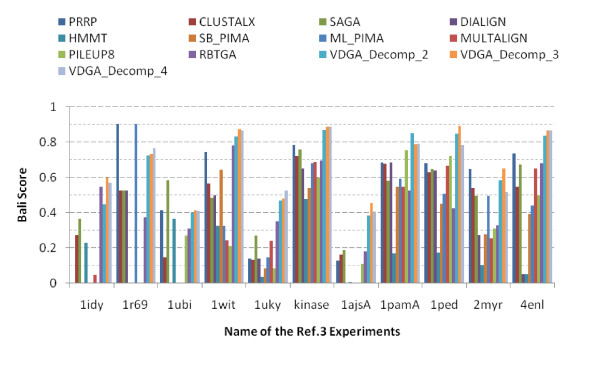
**Graphical presentations of the experimental results on reference 3 datasets**.

We have also compared our results with RBT-GA. We have considered all of the 34 datasets and their approximate results as reported in the RBT-GA paper [35]. The summary of the experimental results of references 2 and 3 are presented in Table 7 and Table 8 and are plotted in Figure 13 and Figure 14 respectively.

**Table 7:** Experiments on Reference 2 Datasets of BAliBase

**Reference No**.	Name of Dataset	PRRP	CLUSTALX	SAGA	DIALIGN	HMMT	SB_PIMA	ML_PIMA	MULTALIGN	PILEUP8	RBT-GA	VDGA_Decomp_2	VDGA_Decomp_3	VDGA_Decomp_4
Ref. 2	1aboA	0.256	0.65	0.489	0.384	0.724	0.391	0.22	0.528	0.000	**0.812**	0.723	0.791	0.679
	
	1idy	0.37	0.515	0.548	0.000	0.353	0.000	0.000	0.401	0.000	**0.997**	0.981	**0.992**	0.992
	
	1csy	0.35	0.154	0.154	0.000	0.000	0.000	0.000	0.154	0.114	0.735	0.731	**0.885**	0.831
	
	1r69	0.675	0.675	0.475	0.675	0.000	0.675	0.675	0.675	0.45	0.9	0.859	**0.934**	0.874
	
	1tvxA	0.207	0.552	0.448	0.000	0.276	0.241	0.241	0.138	0.345	0.891	0.944	**0.974**	0.944
	
	1tgxA	0.695	0.727	0.773	0.63	0.622	0.678	0.543	0.696	0.318	0.835	0.867	**0.878**	0.850
	
	1ubi	0.056	0.482	0.492	0.000	0.053	0.129	0.129	0.000	0.000	**0.795**	0.732	0.778	**0.794**
	
	1wit	0.76	0.557	0.694	0.724	0.641	0.469	0.463	0.5	0.476	0.825	**0.875**	0.815	0.774
	
	2trx	0.87	0.87	0.87	0.734	0.739	0.85	0.702	0.87	0.87	0.982	0.959	**0.986**	**0.986**
	
	1sbp	0.231	0.217	0.374	0.043	0.214	0.043	0.054	0.186	0.177	**0.778**	0.782	0.772	**0.778**
	
	1havA	0.52	0.48	0.448	0.000	0.194	0.259	0.238	0.5	0.493	0.792	**0.884**	0.846	0.884
	
	1uky	0.351	0.656	0.476	0.216	0.395	0.256	0.306	0.585	0.562	0.625	0.845	**0.891**	0.872
	
	2hsdA	0.404	0.484	0.498	0.262	0.423	0.39	0.561	0.593	0.278	0.745	**0.856**	0.829	0.742
	
	2pia	0.767	0.752	0.763	0.612	0.647	0.73	0.695	0.765	0.766	0.730	0.847	**0.850**	0.839
	
	3grs	0.363	0.192	0.282	0.350	0.141	0.183	0.211	0.192	0.159	0.755	0.717	0.751	**0.781**
	
	Kinase	0.896	0.848	0.867	0.692	0.749	0.755	0.651	0.83	0.799	0.712	0.825	**0.888**	0.812
	
	1ajsA	0.227	0.324	0.311	0.000	0.242	0.000	0.000	0.311	0.227	0.892	**0.906**	0.905	0.902
	
	1cpt	0.821	0.66	0.776	0.425	0.388	0.184	0.277	0.777	0.688	0.584	0.869	0.812	0.853
	
	1lvl	0.772	0.746	0.726	0.783	0.539	0.62	0.688	0.614	0.678	0.567	0.803	**0.819**	0.816
	
	1pamA	0.711	0.761	0.623	0.576	0.53	0.393	0.386	0.566	0.702	0.66	0.857	**0.863**	0.853
	
	1ped	0.881	0.834	0.835	0.773	0.696	0.651	0.647	0.741	0.749	0.78	0.935	**0.947**	0.943
	
	2myr	0.582	0.904	0.825	0.84	0.443	0.727	0.75	0.894	0.786	0.675	0.806	**0.830**	0.808
	
	4enl	0.668	0.375	0.739	0.122	0.213	0.096	0.092	0.384	0.224	0.812	0.890	0.889	**0.899**

Average Score		0.541	0.583	0.586	0.384	0.401	0.379	0.371	0.517	0.429	0.777	0.848	**0.866**	0.849

**Table 8:** Experiments on Reference 3 Datasets from BAliBase

**Reference No**.	Name of Dataset	PRRP	CLUSTALX	SAGA	DIALIGN	HMMT	SB_PIMA	ML_PIMA	MULTALIGN	PILEUP8	RBT-GA	VDGA_Decomp_2	VDGA_Decomp_3	VDGA_Decomp_4
Ref. 3	1idy	0.000	0.273	0.364	0.000	0.227	0.000	0.000	0.045	0.000	**0.546**	0.446	0.599	0.569
	
	1r69	**0.905**	0.524	0.524	0.524	0.000	0.000	**0.905**	0.000	0.000	0.374	0.724	0.733	0.765
	
	1ubi	0.415	0.146	**0.585**	0.000	0.366	0.000	0.000	0.000	0.268	0.31	0.398	0.414	0.410
	
	1wit	0.742	0.565	0.484	0.500	0.323	0.645	0.323	0.242	0.210	0.78	0.833	**0.873**	0.867
	
	1uky	0.139	0.130	0.269	0.139	0.037	0.083	0.148	0.241	0.083	0.35	0.469	0.481	**0.526**
	
	kinase	0.783	0.720	0.758	0.650	0.478	0.541	0.682	0.688	0.599	0.697	0.870	**0.890**	0.887
	
	1ajsA	0.128	0.163	0.186	0.000	0.006	0.000	0.000	0.000	0.110	0.18	0.383	**0.453**	0.408
	
	1pamA	0.683	0.678	0.579	0.683	0.169	0.546	0.590	0.546	0.754	0.525	**0.853**	0.788	0.792
	
	1ped	0.679	0.627	0.646	0.641	0.172	0.450	0.507	0.665	0.722	0.425	0.848	**0.893**	0.783
	
	2myr	0.646	0.538	0.494	0.272	0.101	0.278	0.494	0.253	0.310	0.33	0.586	0.651	0.519
	
	4enl	0.736	0.547	0.672	0.050	0.050	0.393	0.438	0.652	0.498	0.68	0.836	**0.866**	**0.866**

Average Score		0.532	0.446	0.506	0.314	0.175	0.267	0.372	0.303	0.323	0.472	0.659	**0.695**	0.672

#### • Performance of VDGA in Reference 2

The 23 datasets in this reference are significantly different in lengths and numbers of their sequences. They also contain what is called "orphan sequences". VDGA performed differently with different datasets. To judge the performance of VDGA with respect to BAliscore, we have compared with SAGA, RBT-GA, PRRP, CLUSTALX, DIALIGN, HMMT, SB_PIMA, ML_PIMA, MULTALIGN and PILEUP8. Table 7 and Figure 13 show that for the 23 test cases, the VDGAs (Decomp_2, Decomp_3 and Decomp_4 together) were successful in finding more accurate solutions than the others in 21 test cases, and RBT-GA was successful in finding better solutions in 3 out of 23 test cases. RBT-GA and VDGA achieved the same best value in one test case. Among the successful 21 test cases, VDGA with Decomp_2 found the best BAliscore in 6 test cases, VDGA with Decomp_4 in four test cases and VDGA with Decomp_3 successfully found better solutions in 11 test cases. In 3 test cases, where VDGA could not achieve the best solution, it was close to the best solution.

**Figure 15:**
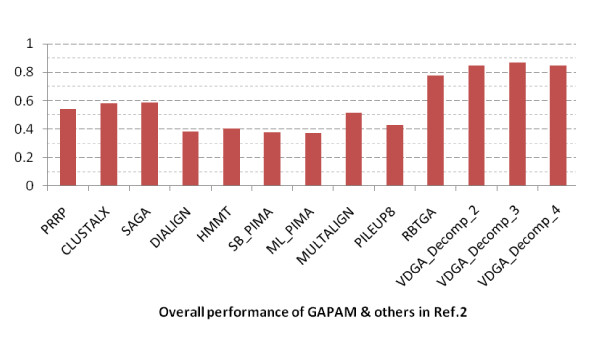
**Overall Performance of all methods in reference 2 datasets**.

The average scores are also shown in Table 7 and are plotted in Figure 15. This figure shows that all vertical decompositions achieved higher average accuracy than the other methods considered in this section. Of those, VDGA_Decomp_3 achieved the highest average accuracy, as it performed better for almost all test cases in reference 2. The average CS score of VDGA for reference 2 was 0.814.

#### • Performance of VDGA in Reference 3

Reference 3 contains sub-groups of sequences where the residue identities between groups are less than 25%. In this paper, we considered 11 test cases out of 12, and the experimental results that are presented in Table 8 and Figure 14 show that VDGA found more accurate MSAs in 9 test cases, where VDGA_Decomp_2 was best in 1 test case, Decomp_3 in 5 and Decomp_4 in 2 test cases. VDGA_Decomp_3 and 4 found the same highest score in one test case. Whereas SAGA was successful in finding the best score in one, PRRP in one and ML_PIMA in one test case. PRRP and ML_PIMA found the same solution for one test case (1r69). Figure 14 shows that for some test cases, most of the methods could not find any similarities in their solutions, in comparison to the reference alignments. Therefore, these methods received zero score. PRRP and ML_PIMA achieved the same best score in one test case, but both received a zero score for another test case. However, VDGA did not obtain any zero score.

**Figure 16:**
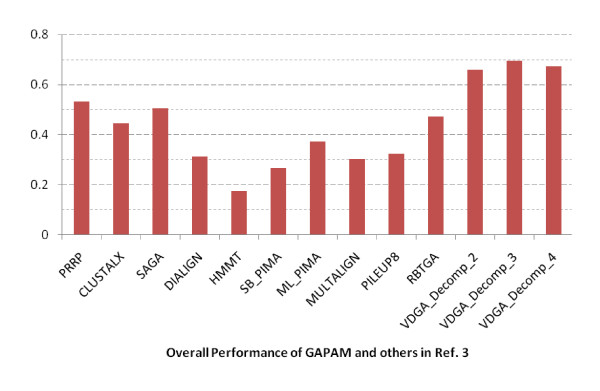
**Overall performance of all methods in reference 3 datasets**.

The overall performance of all the methods for this reference is presented in Figure 16. Although the VDGA method did not achieve high accuracy solutions in some test cases, the average performance of this method with three decompositions are clearly better than the others as shown in Figure 16. The average scores of VDGA_Decomp_2 and Decomp_4 are higher than other methods, while VDGA_Decomp_3 achieved the highest average score. Therefore, we can conclude that the overall performance of VDGA in reference 3 is also better than the other methods mentioned earlier. The average CS score of our VDGA approach for the RBT-GA selected datasets for reference 3 was 0.524.

### Statistical Analysis

To study the difference between any two stochastic algorithms in a more meaningful way, we have performed statistical significant testing. We have chosen a non-parametric test, Wilcoxon Signed Rank test [50], as it allows us to judge the difference between paired scores when it cannot make the assumption required by the paired-samples *t *test, such as that the population should be normally distributed. The results based on the best found solutions of VDGA are presented in Table 9, where W (= W_+ _or W_-_) is the sum of ranks based on the absolute value of the difference between two test variables. The sign of the difference between two independent samples is used to classify cases into one of two samples: differences below zero (negative rank W_- _), or above zero (positive rank W_+ _). As a null hypothesis, it is assumed that there is no significant difference between two samples. The alternative hypothesis is that there is a significant difference in the fitness values of the two samples. Hence, if the hypothesis test rejects the null hypothesis, then there is a significant difference, otherwise there is no significant difference. The number of test problems is *N *= 26 and 34 for MSA-GA and RBT-GA respectively, and we have used the 5% significance level. Based on the test results/rankings, we assigned two words ('yes' for P < = 0.05 or 'no' for P > 0.05 ) for the comparison of any two algorithms (as shown in the fifth column), where 'yes' means that the VDGA algorithm (with Decomp_3) is significantly different and better than the second, 'no' means that this is worse and there is no significant difference between the two algorithms, and 'same' means there is no significant difference and VDGA performed the same as the second. We tested for significant with the BAliscore corresponding to the best found WSPM scores produced by VDGA, in comparison to the published BAliscore results of the other methods.

**Table 9:** The Wilcoxon Signed Ranks Test results for the VDGA_Decomp_3 and other methods (With Respect To the BAliscore based on negative ranks)

Algorithm	W+	W-	Ties	Z	P	VDGA_Decomp_3 is Significant if P < 0.05	Hypothesis test Decision (null hypothesis)
**MSA-GA**	23	3	0	-3.886	0.000	yes	Reject

**MSA-GA w/prealign**	22	4	0	-3.391	0.001	yes	Reject

**ClustalW**	22	4	0	-3.518	0.000	yes	Reject

**PRRP**	30	4	0	-4.693	0.000	yes	Reject

**CLUSTALX**	33	1	0	-5.035	0.000	yes	Reject

**SAGA**	33	1	0	-4.864	0.000	yes	Reject

**DIALIGN**	33	1	0	-5.069	0.000	yes	Reject

**HMMT**	34	0	0	-5.086	0.000	yes	Reject

**SB_PIMA**	34	0	0	-5.086	0.000	yes	Reject

**ML_PIMA**	33	1	0	-5.001	0.000	yes	Reject

**MULTALIGN**	33	1	0	-5.035	0.000	yes	Reject

**PILEUP8**	34	0	0	-5.086	0.000	yes	Reject

**RBTGA**	28	6	0	-4.599	0.000	yes	Reject

**VDGA_Decomp_2**	44	10	2	-4.577	0.000	yes	Reject

**VDGA_Decomp_4**	38	14	4	-3.534	0.000	yes	Reject

In this comparison, we have considered only Decomp_3 with VDGA, as we have found from the preceding experiments that Decomp_3 is the best for VDGA. Therefore, to test the significance we have considered only VDGA_Decomp_3. In Table 9, it shows that there is a significant difference when VDGA_Decomp_3 is compared with MSA-GA, MSA-GA w/prealign and CLUSTAL W for the dataset used in MSA-GA, and when comparing VDGA_Decomp_3 with PRRP, CLUSTALX, SAGA, DIALIGN, HMMT, SB_PIMA, ML_PIMA, MULTALIGN, PILEUP8 and RBT-GA for the dataset used in RBT-GA, as indicated by the hypothesis test decision and the significance values. VDGA_Decomp_3 is also significantly different than VDGA_Decomp_2 and VDGA_Decomp_4 for all the test cases. From the experimental observations, it is clear that VDGA is significantly better according to the Wilcoxon Signed Ranks test.

## Conclusions

In this paper, a new GA based algorithm with Vertical Decomposition (VDGA) has been proposed to solve multiple sequence alignment problems. This approach works with the solution of a guide tree. To generate an initial population, two mechanisms are used. To assess the performance of the algorithm, a number of experiments were carried out for deciding the initial population, the genetic operator, an appropriate set of parameters for GA and of the suitable number of decompositions.

An initial experiment was run to determine the parameters, and from the experimental results, the probability of crossover and mutation was set to 50%-50%. A simple hill climbing method with the standard VDGA initial population was performed to verify the performance of the genetic operators. Moreover, the VDGA method was also run with a randomly generated initial population to judge the performance of the initial generation. To test the performance of the decomposition, this technique was applied on the solution of the guide tree as well as inside the GA. The experimental results showed that the decomposition technique can successfully find better multiple sequence alignments, and also that the optimum number of decompositions for VDGA is 3 (i.e. Decomp_3).

To evaluate our proposed approach, we considered a good number of benchmark datasets from BAliBase 2.0, so as to cover all the test sets of MSA-GA and RBT-GA. The proposed method was optimized based on the Weighted Sum of Pair score. Therefore, the BAliscore corresponds to the best WSPM score. This was used to compare with other methods, as the BAliscore is widely used as the measure of quality/accuracy of multiple sequence alignments. The experimental results showed that VDGA performed better for most of the test cases. Although the solution of VDGA was not the best for some test cases, it was close to the best for those cases. The overall behavior of our proposed method outperformed all of the other methods considered in this paper. VDGA performed better than the others mainly because of our proposed initial generation, the genetic operators, the operators setting, and most importantly its decomposition technique.

After statistical and experimental analysis, we can safely conclude that the proposed method, VDGA with Decomp_3, can be considered as an effective method for solving multiple sequence alignment problems.

Future studies intend to extend the experiments and to find a more efficient way for decomposition of longer and many sequences. We also want to optimize VDGA with different scoring schemes, such as the recent Log Expectation scoring function [17], as well as the consistency-based objective functions of COFFEE [52] and T-COFFEE [12], in order to test its performance.

## Authors' contributions

FN was responsible for conception, design, implementation, testing and drafting of the VDGA. RS and DE were responsible for revising this proposed concept critically and hence for important intellectual content. All authors have read and approved the final manuscript. The first author contributed 50 percent of this work. The other authors contributed 25 percent each.
